# Research progress on the correlation between cataract occurrence and nutrition

**DOI:** 10.3389/fnut.2024.1405033

**Published:** 2024-07-01

**Authors:** Yi Zhang, Xiang Qin, Tianyu Xu, Fangjing Chu, Beihui He

**Affiliations:** The First Affiliated Hospital of Zhejiang Chinese Medical University (Zhejiang Provincial Hospital of Chinese Medicine), Hangzhou, China

**Keywords:** cataract, nutrition, oxidative stress, insulin resistance, micronutrients, inflammatory factors

## Abstract

Cataract is a common eye disease characterized by lens opacity, leading to blurred vision and progressive blindness of the eye. Factors affecting the development of cataracts include nutrition, oxidative stress, micronutrients and inflammatory factors, and also include genetics, toxicity, infrared exposure, hyperuricemia, and mechanical injuries. Among the nutritional factors, a balanced diet, vegetarian diet, dairy products and vegetables are protective against cataracts; high-sodium diet, high intake of carbohydrates and polyunsaturated fatty acids may increase the risk of cataracts; and increased intake of proteins, especially animal proteins, may prevent nuclear cataracts. Intake of antioxidants such as β-carotene, lutein, or zeaxanthin is associated with a reduced risk of cataracts. Minerals such as zinc, selenium, calcium and sodium have also been associated with cataract development. Oxidative stress plays an important role in the development of cataracts and is associated with several antioxidative enzymes and biomarkers such as glutathione (GSH), superoxide dismutase (SOD), malondialdehyde (MDA) and 4-hydroxynonenal (4-HNE). Insulin resistance is also an essential risk factor for cataracts, especially in diabetic patients. In conclusion, understanding these influencing factors helps us to better prevent cataracts. And in this article, we will focus on the important factor of diet and nutrition for a detailed discussion.

## 1 Introduction

Cataract is a common eye disease whose typical symptom is the opacity of the lens, which causes blurred vision and even progressive blindness. According to the World Health Organization (WHO), cataracts affect more than 94 million people worldwide (1), indicating that cataracts have become one of the major causes of global vision impairment. Therefore, exploring the risk factors affecting cataract formation is crucial for the prevention and mitigation of this disease. The object of this study is to comprehensively analyze all known influencing factors, including dietary structure, chronic diseases, to deepen the understanding of the pathogenesis of cataract and providing scientific evidences for the development of effective preventive measures. At the same time, this study will also examine some generally proposed influencing factors, such as nutrient intake, oxidative stress, insulin resistance, etc., in order to expand our knowledge in cataract formation. By systematically analyzing these factors, this study hopes to facilitate preventive intervention and mitigation of early cataract formation and provide new thoughts and solutions for clinical practice.

## 2 Correlation of dietary mode and intakes with cataract

### 2.1 Dietary mode

Falkowska et al. (2) pointed out that a balanced diet (mainly based on calorie restriction of vegetables, grains, legumes and fish, and a nutrient pattern of low animal fats), a vegetables diet, “dairy and vegetables,” “traditional diet,” “antioxidants” and “Omega-3 (polyunsaturated fatty acids)” have a significant protective effect on age-related cataracts. A large hospital cohort study conducted by Jee and Park (3) assessed lifestyle-related risk factors. Metabolic syndrome (MS) was positively associated with age-related cataract (ARC). In addition, they found that individuals with hyperglycemia and low density lipoprotein (LDL) cholesterolemia were more prone to ARC, and plasma glucose and HbA1c concentrations increased 1.50 and 1.92-fold, in patients with MS exhibiting ARC risk. High carbohydrate intake increased the risk of ARC by 1.4-fold, and high fat (≥15%), protein and calcium intake reduced the risk of ARC by 0.74-fold. Movahedian (4) et al. found a significant and direct relationship between dietary glycemic index, glycemic load, insulin load and cataracts, and speculated that improving the quality of the diet is a key approach to reduce the risk of cataracts.

### 2.2 Carbohydrate intake

Several studies have shown (5, 6) that carbohydrate intake is positively associated with cataract development. The specific mechanism of the reaction is unknown, and it may be that the slow absorption and utilization of glucose by the ocular aqueous humor leads to prolonged exposure of lens proteins to higher concentrations of glucose, causing the occurrence of protein cross-linking, aggregation, and precipitation, which leads to lens opacity and the development of cataracts (7). In addition, carbohydrates can elevate blood glucose concentrations, increasing the risk of cataract development (8).

### 2.3 High fat diet

Lu et al. (9) found that intake of large amounts of linoleic or linolenic acid increases the risk of lens opacity, which may ultimately lead to cataracts, because of the susceptibility of unsaturated fatty acids to lipid peroxidation, which results in the susceptibility of lens epithelial cells to oxidative stress, causing cell damage.

## 3 Correlation of glucose metabolism with cataract

### 3.1 Association of insulin resistance with cataract

Guo (10) found that in patients with diabetic cataract, high levels of fasting plasma glucose (FPG), glycated hemoglobin (HbA1c), and insulin resistance index (HOMA-IR) were negatively correlated with visual acuity, and positively correlated with intraocular pressure (IOP) serum levels of interleukin-6 (IL-6), insulin-like growth factor (IGF-1), and vascular endothelial growth factor (VEGF) levels. This suggests that IL-6, IGF-1, and VEGF are important inflammatory factors promoting insulin resistance, and glucose fluctuations in diabetic cataracts and these factors are closely related to oxidative stress injury. It has been noted (11, 12) that NADPH oxidase-mediated oxidative stress and P53 (p53 is a tumor suppressor protein that regulates the expression of a variety of genes that are involved in apoptosis, growth arrest, inhibition of cell cycle progression, differentiation and accelerated DNA repair or senescence in response to genotoxicity or cell stress.) and Bax/Bcl2-mediated apoptosis signaling pathways are involved in the pathogenesis of cataracts in diabetic rats, and Puerarin may exert a therapeutic effect on diabetic cataract rats by improving the state of insulin resistance and downregulating the level of P53 protein (12).

### 3.2 The pathogenesis of diabetic cataract

In diabetic patients, prolonged hyperglycemia leads to excessive accumulation of sorbitol in the lens, increased osmotic pressure, influx of aqueous humor, leading to intracellular edema and fiber breakage, ultimately leading to lens turbidity and further worsening the extent of oxidative stress. It has been proposed (13) that cataract formation usually occurs in the several weeks or months after the initiation of insulin treatment in type I diabetes, and insulin autoantibodies are positive within 3 months of the initiation of insulin therapy, which coincided with cataract formation. Martina et al. (14) found that the development of cataracts in type II diabetes mellitus was closely related to the course of diabetes and various metabolic risk factors, especially the coexistence of poor glycemic control, hypercholesterolemia, diastolic blood pressure and diabetic nephropathy.

Decrease in insulin level or inhibition of galactokinase activity in the patients results in increased blood glucose content and the osmotic pressure within the lens. Lens fibers will be swelling and contain fractures, with increased opacity due to excessive osmotic pressure elevation. Early and timely diagnose is mandatory under this situation (15). After testing the serum levels of HbA1c, FPG, and fasting insulin (FINS) in patients with diabetic cataracts and other cataracts, calculating HOMA-IR, and testing the levels of IGF-1 and IL-6, Tang et al. (15) found that the other cataract patients had lower levels of HbA1c, FPG, HOMA-IR, IGF-1, and IL-6 than the patients with diabetic cataracts. Therefore, they believed that these indicators can assist in determining the condition. Guo et al. (10) yielded similar results and also mentioned that the IOP of diabetic cataract patients was higher than other cataract patients. Cai et al. (16) analyzed the relationship between postoperative insulin resistance and changes in inflammatory factor levels and quality of vision in cataract patients with glaucoma. It was mentioned that the patient’s preoperative objective scatter index (OSI) was negatively correlated with insulin sensitivity index, that is, the degree of insulin resistance correlated with the degree of lens opacity. Suryanarayana et al. (17) showed that impaired glucose tolerance (IGT), which is suggestive of insulin resistance, affects cataract development in rat model experiments.

## 4 Correlation of fat metabolism with cataract

### 4.1 Correlation of hyperlipidemia and cataract

Yin et al. (18) proposed that hyperlipidemia is the main factor for increased lens density in patients with age-related nuclear cataract. They found that serum total cholesterol (TC), triglyceride (TG), low-density lipoprotein cholesterol (LDLC) levels in age-related nuclear cataract were correlated with lens density. Zhang et al. (19) suggested that hyperlipidemia promotes the development of galactose cataract to some extent based on data derived from rats experiments and suggested that cataract formation may be involved in the oxidative stress response. The hyperlipidemic cataract group had reduced GSH and higher malondialdehyde contents than the normal cataract group, reflecting reduction of antioxidative capacity and increase of damages caused by free radicals under a hyperlipidemic status (20). This reflects the reduced activity of the antioxidant system in the lens of rats in the hyperlipidemic cataract group, and the increased malondialdehyde content suggests an increased severity of cell attacked by free radicals. Similar results were obtained by Tsutsumi et al. (20) in rats experiments, leading to a conclusion that hyperlipidemia that hyperlipidemia and LDLC are risk factors for the development of diabetic cataracts. In a case–control study conducted by Galeone et al. (21), cataract occurrence was found to associate with hyperlipidemia, central obesity, hypertension and diabetes. Zhang et al. (22) also concluded that age-related cataract patients are often afflicted with hyperlipidemia, hypertension and hyperglycemia. They found that oxidized low-density lipoprotein (oxLDL) can effectively affect lens epithelial cell transcription, leading to differential expression of Rho signaling and ATP1B1, which may be involved in cataract formation.

However, Jiang et al. (23) proposed a different viewpoint, with data showing that only the decrease in HDLC content increased the risk of age-related cataracts in middle-aged and elderly people, and there was no correlation between TC and TG, and they did not discuss LDL. This is inconsistent with the previous view. However, at present, there is not enough research to support the inevitable relationship between hyperuricemia and the development of cataract.

### 4.2 The relationship between MAFLD and cataract

In a Korean study (24), it was found that metabolism-associated fatty liver disease (MAFLD) was more closely associated with cataracts than non-alcoholic fatty liver disease (NAFLD). MAFLD may be a combined risk factor for cataracts, with significant correlations with all cataract subtypes because cataracts can be caused by various metabolic and inflammatory conditions, and MAFLD covers alcohol-induced cirrhosis and various metabolic diseases. In addition, hepatic factors and the signaling proteins secreted by the liver can reach the eye and cause oxidative stress and inflammation.

## 5 Correlation of mineral metabolism and cataract

Minerals are also closely related to cataracts, and nutritional dystrophy (lack of macroelement and microelement, insufficient oxygen supply, and reduced hemoglobin) can lead to the development of nuclear cataracts (25).

### 5.1 Zinc

The content of zinc in ocular tissues, especially in retina, is significantly higher than in other tissues. One study found that combining zinc and vitamin A as a nutrient supplement reduced the incidence of cataracts (26). It can be assumed that oxidative damages cause alterations in the structure of lens proteins, while antioxidant metalloenzymes relied by zinc, such as SOD, protect the lens (27).

### 5.2 Selenium

Selenium acts as a functional heteroatom in peroxidases and plays an important role in the metabolism of H_2_O_2_ and other peroxides, effectively preventing free radical production and damages to tissues. If the amount of selenium exceeds the amount required for the synthesis of proteins containing selenium, a redox cycle is initiated, which promotes oxidative damage and ultimately induces cataract development in laboratory animals (28). In addition, Dawczynski (29) et al. found that the amount of selenium in the lens increases with increasing lens opacity, and the amount of selenium in the lens is higher in the mature stage of cataract than in other stages, whereas serum selenium shows the opposite change. But the exact mechanism is still unknown.

### 5.3 Calcium

When the concentration of calcium ions in the aqueous humor deviates from the normal value, either too high or too low, it will lead to the development of lens opacity, which is named as hypercalcemic cataract and hypocalcemic cataract, respectively. Delamere et al. (30) constructed a model of hypocalcemic cataract in rabbits by feeding a low-calcium diet and divided the hypocalcemic cataracts into three stages, including posterior subcapsule punctate opacity, dense opacity, and extension to the superficial anterior cortical layer, and suggested that aqueous humor calcium can be inferred from the serum calcium. Yang et al. (31) incubated rabbit lens in different concentrations of CaCl_2_ solution for 36H and found that all the rabbit lenses could develop hypercalcemic cataracts with complete cortical turbidity when the CaCl_2_ concentration in the culture solution was ≥30 mmol/L. The homeostasis of calcium ions plays an important role in maintaining lens transparency. When the cell membrane is damaged, large amounts of calcium ions from the aqueous humor move in to the lens and activate calpain, leading to the degradation of numerous important structural proteins (31).

### 5.4 Sodium

High intake of sodium is also a factor affecting cataracts (32). When the lens cell membranes are damaged by oxidative stress, the function of Sodium-Potassium Pump that maintains the normal low Na + and high K+ concentrations is altered in the cells, which increases the permeability of sodium ions and the concentration of sodium ions in the lens, exacerbating lens opacity.

## 6 Correlation of vitamins with cataract

Vitamin A, vitamin C, and vitamin E have been associated with a reduced risk of ARC too. Vitamin C, also named as ascorbic acid, is a strong antioxidant in the body. Its remarkable antioxidant properties have been negatively correlated with the development of cataracts. It can react rapidly with free radicals such as O-, HOO-, and OH- to generate semi-dehydroascorbic acid and be beneficial to the scavenging of single oxygen (33). Ge et al. (34) suggested that the antioxidant efficacy of vitamin C in organisms far exceeds that of the *in vitro*. It may be attributed to its action *in vivo* through a range of indirect mechanisms rather than direct scavenging oxygen radicals. So, increasing the intake of vitamin C can be considered as an effective strategy to improve the antioxidant capacity of the organism and help to prevent cataracts.

In a streptozotocin (STZ)-induced oxidative stress model in senile diabetic rats (35), dietary vitamin C and E supplementation alleviated oxidative stress and increased the antioxidant levels of the lens. The mechanism may be that reduced GSH synergises with antioxidant vitamins to resist oxidative stress. Vitamin E transfers its hydrogen to superoxide radical of polyunsaturated fatty acids, breaking the radical chain reaction and preventing the peroxidation of polyunsaturated fatty acids in the cellular and subcellular membranes. These vitamins can also directly scavenge ROS and increase the activity of antioxidant enzymes.

There is a considerable controversy regarding the effect of vitamin E on cataract formation. Increasing dietary intake of food rich in vitamin E and supplying vitamin E pill to the population may reduce the risk of nuclear cataract development (36), but most studies do not show results in agreement with this hypothesis, especially in studies regarding the role of vitamin E in cortical cataracts (37).

The literature published by Spector et al. (38) mentioned that nutrient interventions may be a solution to reduce cataract risk. They showed that increasing intake of vitamin C, lutein, xanthines and dietary fruit can reduce the incidence of age-related cataracts. Lawrenson and Grzybowski (39) have also shown that a diet containing antioxidants can delay the progression of cataracts. Nutrient factors, particularly vitamins with antioxidant properties, are thought to play a protective role in cataract development and progression. Vitamin A, niacin, riboflavin, thiamin, folic acid and vitamin B12 appear to have a protective effect, either alone or as components of multi-vitamin preparations (26, 40).

## 7 Analysis of other nutritional factors related to cataract

### 7.1 The relationship between oxidative stress and cataract

Oxidative stress is a state of imbalance between oxidative and antioxidative effects in the body, with more oxidative stress due to higher oxidative effects. Oxidative stress plays an important role in the pathogenesis of all types of cataracts. The lens derives its energy mainly from the glycolysis, so the lens inevitably generates a large number of oxygen radicals, such as superoxide anion (O^2^-), hydroxyl radical (OH-), and hydrogen peroxide (H_2_O_2_) during metabolism, causing oxidative stress on the membranes and proteins in the lens, affecting lens transparency in different degrees. If the degree of oxidation exceeds the regulatory capacity of the antioxidant system, lens damage occurs, leading to cataract.

#### 7.1.1 Mechanisms of oxidative stress-induced cataract

In almost all types of cataract lens, the amount of GSH decreases with increasing lens opacity, so deficiency of GSH is thought to be one of the causes of cataract. Antioxidant enzyme systems, such as SOD, facilitate to scavenge O^2^-, protect cells from oxidative stress damage, and maintain redox balance in the lens. Zhou et al. (41) have shown that H_2_O_2_ can impair the antioxidant system of the lens, reducing GSH content and decreasing SOD viability to promote the occurrence of cataract.

#### 7.1.2 Methods to mitigate the oxidative damage

The extract of concha haliotidis significantly reduced H_2_O_2_-induced cataract formation, and its mechanism is to protect lens SOD activity, maintain GSH content, and reduce lens epithelial cell damage (42). In addition, it was found (43) that quercetin improved selenite-induced cataract in rats by enhancing the Nrf-2/HO-1 signaling pathway, through inhibition of oxidative stress. Kang et al. (44) pointed out that nitric oxide synthase (NOS) mRNA was positively correlated with the representative level of oxidative stress in the organism, SOD, in the lens of cortical cataract patients. They further proposed that NOS mRNA is also a factor involved in the process of oxidative stress in patients with cortical cataracts and has a certain effect on the progression of cataracts. It has been shown in the literature (45, 46) that nuclear factor erythroid 2-related factor 2/Kelch-like ECH-associated protein 1 (Nrf2-keap1) is the main mechanism of resistance to oxidative stress, and demethylation of keap1 promoter activates the expression of Keap1 protein, which promotes the degradation of Nrf2 proteasome and attenuates Nrf2 activity, leading to the reduction of resistance to oxidative stress and ultimately causing the cataract occurrence. In addition to Nrf2-keap1, Song et al. (47) proposed that blood suppressor II (BDS-II) inhibits oxidative stress-induced cataract by suppressing oxidative stress-induced reduction of GSH levels and lens epithelial cell death. BDS-II is a blocker of Kv3 channel (voltage gated potassium ion channels), which mainly blocks the oxidation-sensitive channel Kv3.4 to resist oxidative stress.

In normal conditions, Nrf2 is in the cytoplasm, and keap1 protein can maintain the stability of Nrf2 in the cytoplasm. When oxidative stress causes excessive generation of oxygen free radicals, keap1 protein releases Nrf2 through the release of zinc ions, followed by the entrance of Nrf2 into the nucleus to bind to the ARE anti-oxidative stress element sequence, and activates gene transcription downstream of the ARE region, which then initiates the oxidative stress response and reduces the oxidative stress.

Antioxidants have been promoted to delay or prevent cataracts due to their ability to reduce oxidative damage. In a dose–response meta-analysis (48), increasing 5 mg β-carotene daily reduced 10% risk of ARC, and increasing 10 mg lutein or zeaxanthin daily reduced 19% risk of ARC. Lutein and zeaxanthin concentrate in the eye more than any other nutrients and are the only nutrients that have the ability to block blue light and ultraviolet like sunglasses. Blue light and ultraviolet are the primary factors to eye fatigue, blurred vision, and eventually cataracts. Lutein and zeaxanthin are also potent natural antioxidants that resist the destruction of free radical to the eyes. Ma (49) pointed that dietary intake of flavonols (quercetin and isorhamnetin) is associated with the development of age-related cataracts, and increasing dietary intake of quercetin and isorhamnetin may result in a lower risk of age-related cataracts.

## 8 Dietary inflammation index

Shivappa et al. (50) found that eating red meat, sugary drinks, and high-fat dairy products increased the dietary inflammatory index (DII), and higher DII is more likely to be inflammatory. However, the mechanism between DII and the development of cataracts is unclear. One of the possible mechanisms is that pro-inflammatory foods can increase the effects of pro-inflammatory factors (IL-1, IL-6, TNFα) on the transcription of acute-phase proteins, which leads to increased mitosis and collagen synthesis in lens epithelial cells, triggering cataracts. Studies (51) found differences in the expression of inflammatory factors at different ages, which may be related to the development of immune cells in the uvea stroma. This hypothesis can explain that EGF, IL-3, IL-8, and MCP-1 are positively corrective with age. Among the various pro-inflammatory factors, TNFα can promote the production of extracellular matrix (ECM) and regulate the proliferation and differentiation of lens epithelial cells, but the roles of other factors in cataract formation need to be further investigated.

## 9 Prospects and outlook

**Figure 1:**
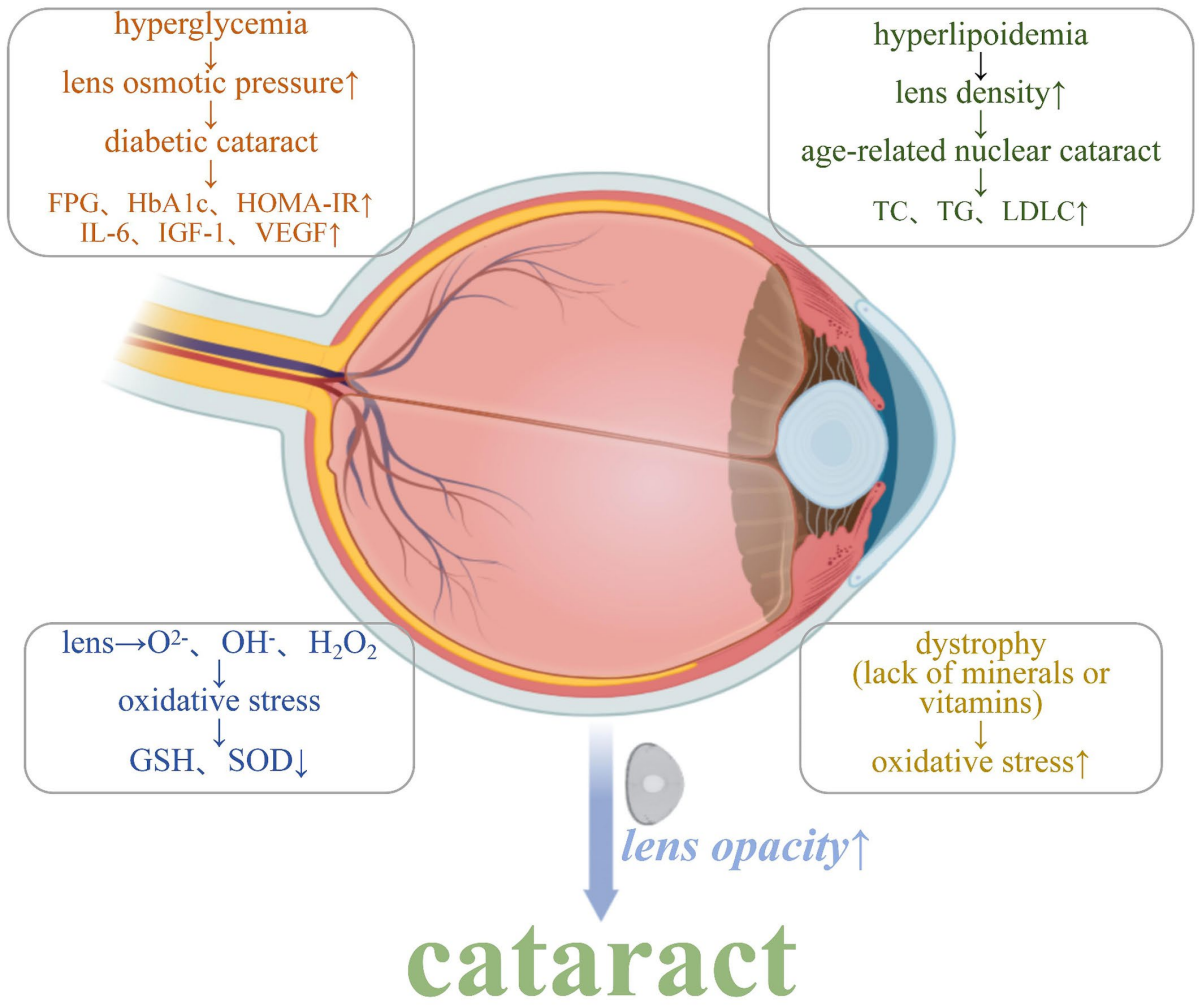
Mechanisms that induce cataract development.

Cataracts have a serious impact on people’s quality of life and need to be treated early, but the occurrence is associated with various factors that make it difficult to prevent. Therefore, continuing and deep studies are necessary to better prevent and to develop novel treat regimen for cataracts. In studying the relationship between dietary nutrition and cataract development, Table 1 highlights key nutrients associated with cataract risk. Understanding their roles in maintaining ocular health provides insights into potential preventative measures. Figure 1 illustrates the mechanisms that lead to cataract formation, including oxidative stress, inflammation, and apoptosis. The followings are some suggested actions that can be taken to fight against cataract formation.

**Table 1:** Nutrients related to the cataract.

Nutrients	Dietary structure	Relativity	Influencing mechanism	Reference
Carbohydrate	high carbohydrate diet (rice, noodles and other staple foods)	Carbohydrate intake was positively associated with cataract development.	① The slow absorption and utilization of glucose in the aqueous humor results in prolonged exposure of lens proteins to high concentrations of glucose, leading to protein cross-linking, aggregation, and precipitation that ultimately cause lens opacity and cataract formation.	(5–8, 52–56)
			② Carbohydrate can also increase blood glucose concentration and increase the risk of cataract.	
	high-sugar diet	Glucose metabolism influences the cataract development.	① The fluctuation of blood glucose in diabetic patients leads to oxidative stress response, causing cataract.	(10–16, 57)
			② In diabetic patients, long-term hyperglycemia leads to excessive accumulation of sorbitol in the lens, increased osmotic pressure, and aqueous humor inflow. Osmotic changes lead to intracellular edema and fiber breakage, which eventually makes the lens become turbid and oxidative stress further worsens.	
			③ In diabetic cataract patients, the increasing expression of apoptosis-related factors in lens cells leads to lens opacity.	
			④ In high glucose environment, the lens capsule is damaged, which increases the permeability and loses barrier function, resulting in excessive water absorption of the lens and fiber swelling, fracture, and turbidity.	
			⑤ There is a correlation between the degree of insulin resistance and the degree of lens opacity.	
Protein	high-protein diet	Higher intake of protein, especially animal protein, can reduce the incidence of cataract.	Animal proteins are complete proteins that contain all the amino acids necessary for the human body. Moderate intake of animal protein can provide the specific amino acids needed by the lens to maintain normal renewal and repair of lens proteins. For example, animal viscera are rich in selenoprotein and selenium is an essential trace element for human body. Selenium has an inhibitory effect on oxidative damage of eye lens, and appropriate supplementation of selenium can reduce the incidence of cataract.	(6, 58)
Lipid	high-lipid diet	High blood lipids increase the risk of cataracts.	① Unsaturated fatty acids are prone to lipid peroxidation, leading to oxidative stress in lens epithelial cells, causing cell damage and increasing the risk of lens opacity.	(5, 6, 9, 19–24, 59–62)
			② The activity of antioxidant system is decreased in hyperlipemia.	
			③ Oxidized low-density lipoprotein can effectively affect the transcriptional expression of lens epithelial cells, leading to differences in the expression of Rho signal transduction and ATP1B1, which may increase the incidence of cataract.	
			④ High-fat environment is prone to metabolism-related fatty liver, and liver factors and signal proteins secreted by the liver can also reach the eyes, causing oxidative stress and inflammation.	
Minerals	zinc	Zinc can reduce the incidence of cataract.	Oxidative damage can cause structural changes of lens proteins, and Zinc-dependent antioxidant metalloenzymes such as superoxide dismutase (SOD)， can protect the lens.	(26, 27, 63–67)	
	selenium	Selenium has two sides to cataract occurrence.	Appropriate intake of selenium can effectively prevent the production of free radicals and damage to tissues, relieve oxidative stress, and delay the production of cataracts.	(28, 29, 68–74)	
			When selenium concentration exceeds the amount required for selenium-containing protein synthesis, the redox cycle starts, which promotes oxidative damage and eventually induces cataract in experimental animals.		
	sodium	High intake of sodium increases the incidence of cataract.	Oxidation can damage the cell membrane of lens, change the function of Na + -K-atpase pump, which maintains normal low Na + and high K+ concentration in cells, to increase the permeability of sodium ions and the concentration of sodium ions in the lens, aggravating lens turbidity.	(32, 75–80)	
	calcium	High calcium cataracts	A large amount of calcium ions enter the aqueous humor and activate calpain, leading to the destruction of a large number of important structural proteins and eventually causing cataract.	(81–87)	
		Low calcium cataract	The decrease of parathyroid hormone results in the disturbance of calcium and phosphorus metabolism. The decrease of calcium increases the permeability of the lens capsule, and electrolyte imbalance in the lens affects its metabolism, leading to the occurrence of cataract.	
Vitamin/dietary fiber	vegetables, fruits, and a vegan diet	Vitamin intake can reduce the incidence of cataract.	① Vitamin C, vitamin E, vitamin A and other vitamins with antioxidant properties:	(34–38, 88–97)
			② B vitamins such as lutein, zeaxanthin, etc.:	
			③ β-carotene: It is converted to vitamin A in the body, exerting antioxidant effects and reducing cataract incidence	
Pro-inflammatory factors	inflammatory diet (red meat, sugary drinks, etc.)	Increased dietary inflammatory index may increase the incidence of cataract.	Inflammatory foods can increase cellular inflammatory factors (IL-1, IL-6, TNF-α), affect the transcription of acute phase proteins, which leads to increased mitosis and collagen synthesis of lens epithelial cells, leading to cataract.	(50, 51)

The mechanisms of action of various risk factors need to be further clarified. For example, the specific mechanism of the inflammatory factors TNF-α and IL-6 in cataract development remains to be revealed. By resolving these signaling pathways, new targets can be provided for therapy.

Explore MAFLD and other areas that have received less attention, change the research thinking, and explore the occurrence and mechanism of cataract formation from other perspectives.

Enhance nutrient intervention research. Dietary components, such as the antioxidants vitamin C, E and flavonoids, have a protective effect against cataract formation, and personalized diet can be developed for different populations in the future.

In conclusion, cataract is a complex and multi-factorial disease that requires multidisciplinary cooperation to study pathogenesis deeply. With the advancement of science and technology, it is believed that more effective prevention and therapy can be found in the future to reduce the burden of cataract on human beings.

## Author contributions

YZ: Writing – original draft, Writing – review & editing. XQ: Writing – original draft, Writing – review & editing. TX: Writing – review & editing, Writing – original draft. FC: Writing – review & editing, Writing – original draft. BH: Writing – review & editing, Project administration, Supervision.
